# Exploring Children’s Social and Emotional Representations of the COVID-19 Pandemic

**DOI:** 10.3389/fpsyg.2020.01952

**Published:** 2020-08-12

**Authors:** Nahia Idoiaga, Naiara Berasategi, Amaia Eiguren, Maitane Picaza

**Affiliations:** ^1^Department of Evolutionary and Educational Psychology, University of the Basque Country UPV/EHU, Bilbao, Spain; ^2^Department of Didactics and School Organisation, University of the Basque Country UPV/EHU, Bilbao, Spain

**Keywords:** COVID-19, children, emotions, pandemic, social representation

## Abstract

COVID-19, a new emerging infectious disease (EID), has spread throughout the world, including Europe. Spain, in particular, has witnessed a significant outbreak of the pandemic. All classes have been canceled, and the government has declared a state of emergency, ordering the lockdown and confinement of the entire population. All children in the country have been confined to their homes since March 13 and are not allowed to leave at any time. This population is thus facing the harshest restrictions. Given the vulnerable situation of children, the aim of this research is to understand how they represent and emotionally cope with the COVID-19 crisis. A free association exercise elicited by the word “coronavirus” was completed by 228 children (age range: 3–12 years) from the North of Spain. To analyze the content, we employed the Reinert method with Iramuteq software for lexical analysis. The results revealed that children represent the COVID-19 as an enemy that is being fought by the doctors. Children are afraid and worried about catching the virus, but mainly because they think they can infect their grandparents, and this makes them feel guilty. Moreover, the lockdown situation has produced conflicting emotions in the children. On the one hand, they are scared, nervous, lonely, sad, bored, and angry, but they also feel safe, calm, and happy with their families. These results indicate the need for governments to also consider children in their management of the current situation by placing greater emphasis on social and inclusive policies to help alleviate the possible effects that they may suffer as a consequence of the pandemic and the lockdown. In short, there is a need to address the psychological, educational, social, health, and well-being needs of children.

## Introduction

Children represent only a small percentage of COVID-19 cases (Hamzelou, 2020; Pavone et al., 2020), and the majority of infected children might appear asymptomatic (Cai et al., 2020) or present mild clinical manifestations (Jiao et al., 2020). It might therefore be tempting to assume that, in comparison with adults, children are less vulnerable to this pandemic (Pavone et al., 2020). However, from the beginning of the pandemic, health authorities and politicians have repeatedly pointed out that because of this mild symptomatology, children may play a significant role in spreading the infection. Consequently, in most countries of the world, schools have been closed (United Nations Educational, Scientific and Cultural Organization, 2020) with children confined to their homes. Nevertheless, each country has imposed its own specific rules for children in the lockdown; while in some countries they are allowed to leave their homes to exercise, play sports, or take walks with their parents, in other countries these activities are prohibited (Garcia, 2020).

Spain is currently one of the European countries most affected by COVID-19. Cases began to multiply exponentially and uncontrollably in early March. In view of this situation, all the schools in the country were closed (Sánchez, 2020a), with the Spanish prime minister declaring a state of emergency on 14th March 2020, ordering a mandatory lockdown for all citizens (Royal Decree 462/2020, 2020). In the same speech, the prime minister stated that the rules of this lockdown were very drastic, possibly the most stringent in Europe and even the world (Merino, 2020; Sánchez, 2020b).

In that speech, there was not a single mention of children, even though the rules of the lockdown are particularly harsh for them. Children were forbidden to leave their homes, with Spain along with Italy, being the only European countries where children were not allowed to go out at all (Granda, 2020; Grechyna, 2020). On the 18th of March, however, the government clarified a detail of this law, which permitted single parents (specifically those unable to leave their children in the care of another adult) to leave their homes accompanied by children to purchase groceries and essential items. Further, children were not allowed to use the communal spaces within their buildings, such as a shared terrace or garden (Royal Decree 465/2020, 2020). In Spain, this absolute lockdown for the children lasted 6 weeks, and then, from April 26, they were allowed to go outside, but only for 1 h a day.

Pediatricians, psychologists, and educators have warned of the serious threats that this confinement may pose to children from both a physical and emotional perspective (Grechyna, 2020; Jiloha, 2020; Léon, 2020; The Spanish Children’s Rights Coalition, 2020), stressing that it is essential that children understand what is happening in order to mitigate the damage that this situation may cause them (Dalton et al., 2020; Wang et al., 2020). However, no research has yet been conducted to explore the ways in which children integrate this coronavirus outbreak into their everyday thinking and how they are coping with the psychosocial impact of the crisis.

During the 1980s and 1990s, a considerable body of research focused on children’s understanding of illness (Myant and Williams, 2005). Most of this research was based on Piaget’s theory of development, essentially demonstrating that children have different perceptions of the disease depending on their age and stage of development (Bibace and Walsh, 1980; Banks, 1990; Gillis, 1990; Hergenrather and Rabinowitz, 1991; Carson et al., 1992; Simeonsson et al., 1993; Kury and Rodrique, 1995; Moss-Morris and Paterson, 1995).

However, these cognitive studies, even in their most modern versions (Vacik et al., 2001; Koopman et al., 2004; Myant and Williams, 2005; Piko and Bak, 2006), have failed to address how children understand specific diseases from a common sense standpoint or the ways in which they deal with illnesses on an emotional level. In contrast, the present study is theoretically framed within the Social Representations Theory (SRT; Moscovici, 1961, 1984) because this theory provides a framework for embracing the symbolic meaning that is assigned to diseases in everyday thinking (Joffe, 2003).

Although relatively little work has been conducted with children from the perspective of social representations (Galli and Nigro, 1987; Galli and Fasanelli, 1995; Cagnin et al., 2004; Goodwin et al., 2004), SRT offers an innovative point of view since the function of social representations is to make familiar the things that are unknown or unfamiliar to us (such as the new COVID-19 pandemic; Galli and Nigro, 1987). Thus, a key concern of this theory relates to how knowledge about a new risky phenomenon is transformed from scientific discourse into the common understanding of lay people (Joffe, 2003). Consequently, extensive research has been carried out with regard to specific emerging infectious diseases (EIDs) within this framework (Joffe and Haarhoff, 2002; Joffe and Bettega, 2003; Joffe and Lee, 2004; Washer, 2006; Idoiaga et al., 2017a,b). However, this work has always been conducted from the perspective of adults.

In recent years, social representation research on several EIDs (Wagner-Egger et al., 2011; Idoiaga et al., 2017a), including the COVID-19 pandemic (Eiguren et al., 2020; Idoiaga et al., 2020 a,b^1^^,^^2^), has revealed that recurring emotional patterns can be observed when it comes to dealing with pandemics. First, EIDs are usually represented in terms of heroes, victims, and villains (Wagner-Egger et al., 2011). The heroes are typically the scientific and medical experts who work to beat the disease, while villains are the media and governments (Washer, 2010). The victims are represented as the infected people, particularly those who are defenseless to face the epidemic (Idoiaga et al., 2017b).

However, the representation of risk is not homogeneous throughout society. The SRT also states that in these moments of crisis specific shared ideas emerge among different groups, and also, of course, among children (Wagner and Hayes, 2005; Washer, 2006). Social representations are important in these contexts because they are constructed based on the particular experiences that each group is living through during the pandemic and the information they receive both from the media and through social interactions (Moscovici and Duveen, 2000).

Moreover, research in the field of social representations (Smith and Joffe, 2013) and EIDs highlights the role played by the emotional context in symbolic thought and its relevance for making a topic recognizable and understandable (Höijer, 2010). In fact, the work carried out so far has revealed that in modern societies there are recurring emotional patterns that emerge in response to the threat of EIDs, with fear being very prominent, along with anger and emotional fatigue (Joffe, 2011; Sherlaw and Raude, 2013; Idoiaga et al., 2017a,b). In the case of children, it has been warned that the lockdown imposed in response to COVID-19 could generate feelings of fear, worry, sadness, or stress (Jiao et al., 2020; Jiloha, 2020; Wang et al., 2020) and that understanding children’s reactions and emotions is essential to properly address their emotional needs (Jiao et al., 2020; Jiloha, 2020).

Given these considerations, it is of critical importance to identify how children understand this health crisis in order to develop strategies and tools that, by taking into account their concerns, will ultimately help them to overcome these unprecedented circumstances. Thus, the main goal of this article is to study how children understand or represent the COVID-19, while observing their emotional response to the coronavirus pandemic in Spain.

## Design

### Sample

A total of 250 children participated in this study between 30th March and 13th April 2020. The sample was recruited in the Basque Country region located in Northern Spain. Of the sample, 52.21% were girls and 47.79% were boys. The mean age of the participants was 7.14 years (*SD* = 2.57) with an age range of 3–12 years.

As additional information on participating families and with regard to the economic status of the families, most of them (85.7%) have a medium economic status, the rest 8.9% have a low economic status, and the remaining 5.4% have a high economic status. Moreover, most of the parents have a university education 71.2% or a bachelor’s degree 23.2%. And, only 2.8% have a secondary education and the 2.8% have a primary education.

Besides, with regard to children care, most of the parents, 46% said that they shared the childcare tasks, 32.4% said that it is the mother who takes care of the children, 18.0% said that it is the father, and 3.2% said that other people take care of their children. Finally, 36.6% of the families had no outside space (such as a balcony, terrace, or garden) in their homes.

### Data Collection Method

Due to the confinement situation, we decided to access the children through their parents. Questionnaires were sent to all schools in the Basque Country region and the schools were asked to forward these questionnaires to the families. In that email, a document was sent explaining how the study should be carried out and a link to do so. In the explanatory document, it was specified to the parents that this was a free association exercise for their children and that they, the parents, would take the role of interviewers. To carry out the exercise, they had to ask to their children two specific questions: (1) These days we are talking a lot about the coronavirus. When you hear the word coronavirus, what comes to mind, or what do you think? (2) How are you feeling these days because of the coronavirus? The parents were then encouraged to transcribe the exact responses given by their children. The document gave two practical examples of how the exercise should be done and how it should not be done (specifying that no suggestions should be made or that the children’s words should not be paraphrased).

All children participated on a voluntary basis, received information about the procedure of the investigation, and their parents gave their consent before participating in the study. This research has obtained the approval of the Ethics Committee of the UPV/EHU [M10/2020/055].

### Data Analysis Method

The Reinert method using Iramuteq software for lexical analysis (Reinert, 1983, 1990) was employed to analyze the corpus of text. This method has frequently been used for the study of social representations (Lahlou, 2001; Klein and Licata, 2003; Kalampalikis, 2005), confirming that the results obtained agree with those of other methods used in this field of research (Lahlou, 1996). Iramuteq software eliminates problems of reliability and validity in text analysis (Reinert, 1996; Klein and Licata, 2003). Using this method, which follows a descending hierarchical analysis format, the analyst obtains a series of classes and statistical cues in the form of typical words and typical text segments (see Idoiaga et al., 2017a). Specifically, the software identifies the words and text segments with the highest Chi-square values, that is, those words and text segments that best identify each class or idea that the participants have repeatedly mentioned. Once these “classes” have been identified, they are associated with “passive” variables (independent variables). In the present case, the passive variable was the age range, that is, young children (3–5 years), middle-aged children (6–9 years), or old children (10–12 years).

In accord with previous research using the Reinert method (Camargo and Bousfield, 2009), the raw data were entered into the Iramuteq software, and the most significant items of vocabulary in each class were selected on the basis of three criteria: (1) an expected value of the word greater than 3; (2) proof of association of the Chi-square, tested against the class [χ^2^ ≥ 3.89 (*p* = 0.05); *df* = 1]; and (3) the word appears mainly in that class, with a frequency of 50% or more.

Reinert method operations are statistical, transparent, and reproducible until the final stage of interpretation, where the analyst assigns a label to each specific vocabulary set that the software had identified as a lexical world on the basis of co-occurrences and distribution patterns (Schonhardt-Bailey, 2013). Finally, as a complementary analysis, Iramuteq also conducts a lexical similarity analysis. This analysis presents in a graphical format the structure of a corpus, distinguishing between the shared parts and the specificities of coded variables. This allows the link between the different forms in the text segments to emerge. That is, this analysis allows to identifying the words’ co-occurrences, providing information on the words connectivity, and thus helping to identify the structure of a text corpus content. It also allows to identify the shared parts and specificities according to the descriptive variables identified in the analysis (Marchand and Ratinaud, 2012).

## Results

**Figure 1:**
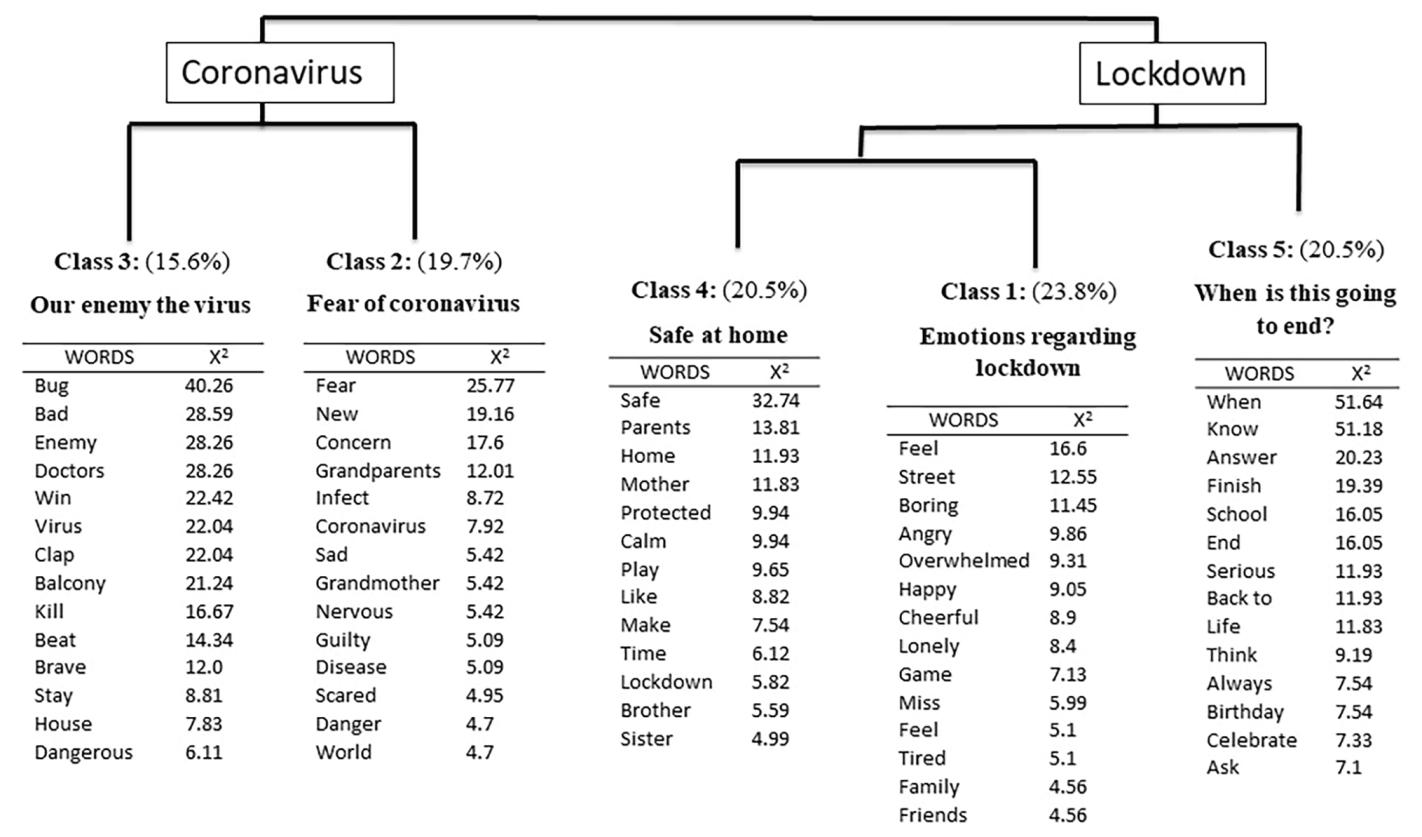
The hierarchical clustering dendrogram of the free association exercise, showing the most frequent words and the words with the greatest association χ^2^(1), *p* < 0.00.

The full corpus contained 12,892 words, of which 1,515 were unique words. Specifically, the descending hierarchical analysis divided the corpus into 211 segments and five classes. The results of this analysis can be observed in Figure 1.

The analysis identified the main ideas held by children regarding COVID-19, elicited through the free association procedure. Each issue or idea is represented by a set of typical words and text segments, which is referred to as a class. First, the results revealed two main branches or themes (composed of different classes), which are referred to as main clusters and labeled as “coronavirus” and “lockdown”. The first main cluster is composed of Classes 3 (Our enemy the virus) and 2 (Fear of coronavirus). The second main cluster is composed of Classes 4 (safe at home), 1 (emotions regarding lockdown), and 5 (when is this going to end?).

Following the hierarchical clustering dendrogram, within the first main cluster describing the coronavirus, the first class to emerge was Class 3, with a weight of 15.6%, which has been labeled as “Our enemy the virus”. Within this class, it can be observed how children describe the COVID-19 with words such as bug, bad, or enemy but they also mention words such as doctors, win, brave, balcony, or clap, praising the work of the doctors to tackle the virus and stressing that what they must do is to stay at home, as can be seen in the characteristic text segments: “It’s a virus but we don’t really know what it is. We have to stay home and beat it because it’s bad and it’s a bug or something that gets into our tummy. In the street the doctors, who are heroes and brave, are going to beat it and that’s why we go out every afternoon to the balcony to clap for them” (*X* ^2^ = 157.75, boy, 4 years); “It’s a bad bug, but we’re going to beat it and the doctors are going to kill it! And get it out of here now!” (*X* ^2^ = 153.59, boy, 5 years); and “It travels by plane and has come here and will not leave. That’s why we have to beat it and to beat it we have to help the doctors and stay home and that’s it, and then everything will be fine” (*X* ^2^ = 145.03, girl, 5 years). This class was mainly elicited by young children (2–5 years; *p* < 0.02).

Within the same “coronavirus” main cluster, the second class emerges, labeled as “Fear of coronavirus” with a weight of 19.7%. This class describes the emotions of fear, concern, sadness, nervousness, or fright created by this health crisis. However, children are more afraid of infecting their grandparents than themselves, even mentioning that they would feel guilty if that happened. The most significant text discourses are: “It’s a virus but since it’s new we’re all a little scared and they talk about it on the radio, on television and everywhere else. It doesn’t hurt children but we can infect our grandparents and that scares me and that’s why we can’t go to their house” (*X* ^2^ = 148.60, girl, 6 years); “Older people say they are afraid but then they go out and buy bread four times a day! I don’t care about those people! I am worried and afraid that something will happen to my grandmother! That’s why I don’t go to her house because if she gets sick I will feel guilty” (*X* ^2^ = 115.88, girl, 12 years); and “The coronavirus is a virus that makes you feel a little afraid but not for yourself, for older people (*X* ^2^ = 98.60, boy, 10 years). This class was mainly elicited by middle-aged children (6–9 years; *p* < 0.01) and old children (10–12 years; *p* < 0.05).

In the second main cluster, classes related to the lockdown situation emerged, including the fourth class (20.5%), which has been labeled as “safe at home.” With words such as safe, protected, calm, home, parents, or mother, children describe how they feel safe and protected at home and are happy with their family, as revealed in the most characteristic segments: “The virus can’t get into my house so I am safe here and I don’t want to go out. Besides, I am happy to play with my family a lot” (*X* ^2^ = 84.83, boy, 7 years) and “I am happy and calm because I like to be with my father and mother and we do many things that I like, and at home we are safe” (*X* ^2^ = 67.64, girl, 5 years).

Within the same main cluster, the first class emerges, labeled as “emotions regarding lockdown” (23.8%). In this class, it is emphasized that children have conflicting emotions during these times. On the one hand, they say they are bored, angry, overwhelmed, tired, and even lonely because they have to stay at home without being able to go out. On the other hand, they also say that they are happy and cheerful being with their family, as can be seen in the characteristic text segments: “Bored because I have to do a lot of homework, sad, and a little lonely because I don’t see my friends or my dog. But also happy because at home we spend more time with my father, mother, and sister and because we clap our hands at the window” (*X* ^2^ = 140.07, boy, 10 years); “I am happy and cheerful but sometimes I get angry because I want to go out and see my friends. It’s a virus that makes me feel angry because it’s a pain in the ass and I can’t decide about anything” (*X* ^2^ = 94.36, girl, 8 years); and “I feel happy when I play with my family. Sometimes I get angry and sometimes I get bored too. If I get angry, I yell and then my mother gets angry” (*X* ^2^ = 74.20, girl, 4 years).

Finally, the fifth class emerges, labeled as “When is it going to end?” (20.5%). Children are very explicit about wanting to know when they will be able to return to school and to their normal life. In addition, many of them are also worried about whether they will still be in confinement on significant dates, for example, on their birthdays. The following are some of the most significant text segments of this class: “I have doubts because I don’t know when this boring confinement is going to end. I want to go back to school and play with my friends” (*X* ^2^ = 144.85, boy, 12 years); and “I want to know when I will go back to school. April 17th is my birthday, I will be 11 and I will have to be at home, and I don’t like it.” (*X* ^2^ = 84.18, girl, 10 years). This class was mainly elicited by the oldest children (10–12 years; *p* < 0.001).

**Figure 2:**
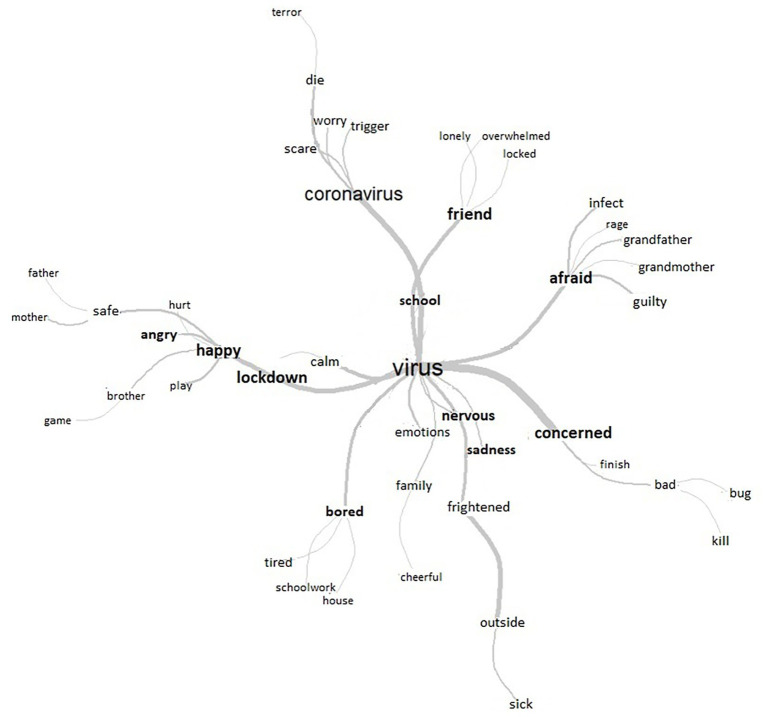
Results of the lexical similarity analysis produced by the sub-corpus of emotions.

Given the wide range of emotions that emerged in the different classes, and in order to analyze these in more depth, we decided to create a Tgen with all the words reflecting emotions and a sub-corpus with these and the associated text segments. This sub-corpus was subjected to a lexical similarity analysis (see Figure 2).

The similarity analysis is interesting to observe the words interconnection as well as the level of relation between them, which rate of co-occurrences between them may be stronger or weaker (Chi-squared test). Based on this analysis, it can be seen more clearly that the coronavirus evokes feelings of fright – and even terror and fear – in the children. This fear is mainly associated with the possibility of infecting their grandparents, along with feelings of guilt. Infants are also concerned about whether the doctors will manage to kill the bad bug (the COVID-19). Moreover, the children are also nervous, sad, and afraid of having to leave their house and are worried about falling ill outside. Therefore, they feel happy, cheerful, calm, and safe at home with their family. However, at the same time, being at home also bores and tires them, particularly when it comes to schoolwork. Finally, this lockdown situation also makes them feel angry.

## Discussion

The findings of this research offer important clues for identifying how children integrate COVID-19 into their everyday thinking. From the voices of the children, the issues that have arisen can be classified into two categories: the coronavirus itself, and the lockdown that has been implemented to control the spread of the virus.

First, coronavirus is represented not only as an enemy, but also as something that could be contagious. Specially from the youngest children’s standpoint, the virus is viewed as something that is very bad and they represent it as a serious bug that is clearly their enemy. In the research carried out so far on COVID-19, and in other work on previous EIDs, other populations (adults and young people) also showed representations of enemies, but interestingly, this enemy or villain was never the disease itself, but the media, the government, or even the citizens who were perceived as behaving in an uncivilized way (Idoiaga et al., 2020b^2^). However, there is agreement regarding the heroes – which are the doctors and healthcare professionals – and also the victims, who are the people most vulnerable to infection (Wagner-Egger et al., 2011; Idoiaga et al., 2020b^2^).

Moreover, older children (the ones from 6 to 12 years) are quite concerned because they know that COVID-19 is highly contagious. In fact, these children expressed their fear, concern, sadness, nervousness, and fright when they were asked about coronavirus. However, they understand the situation well, and most of them are more worried about infecting their grandparents than being infected themselves. However, some of them have expressed that they would feel guilty if someone close to them became infected. This emotion of guilt should be particularly taken into account since in China these feelings have been found to be intrinsically linked to post-traumatic stress (Vidal, 2020). Therefore, it is of vital importance to make it clear to children that they will in no way be blamed if someone close to them becomes infected.

Second, and in relation to the lockdown, we observed the emergence of a sense of security on account of being made to stay at home. They express the idea that for them their house is a safe place and they feel protected at home. However, it should be borne in mind that along with this sense of security, children also express fear of going outside. It is true that at the time at which this research was conducted, children were not allowed to leave their homes under any circumstances. Even so, the street should not be represented as something dangerous or scary because this could have undesirable consequences when the children are eventually permitted to go outside, turning those initial exits into the outside world into traumatic events (Pakpour and Griffiths, 2020).

Returning to the confinement situation, the counter-emotions expressed by the children are remarkable. On the one hand, they are bored, angry, overwhelmed, tired, and even lonely because they have to stay at home without being able to go out. Previous research conducted in China also found that similar negative emotions arise in children regarding the coronavirus lockdown (Jiao et al., 2020; Wang et al., 2020). However, loneliness is a new and striking feeling to emerge in our study. In research with other age groups on COVID-19, loneliness was only aroused in the case of older people (aged over 60; Eiguren et al., 2020). Loneliness is an exceedingly painful experience that is the sum of an unfulfilled need for intimacy and social relationships that are felt to be insufficient or not entirely satisfactory (Berger and Poirie, 1995). Therefore, the emergence of this feeling indicates that peer interaction is extremely important to children (Howes, 2020). That is, they need contact with others such as friends and classmates, and the fact that they feel lonely indicates that they are not receiving the opportunity for such interaction, or at least, not to the extent that is required.

Given the importance of relationships in this growth stage, different strategies must be developed for children to cope with these feelings of loneliness until they have the opportunity to become re-acquainted with friends and classmates. For example, it would be useful to promote socialization strategies from within schools. In other words, in “real life” educational institutions are much more than places, where academic skills are developed; indeed, in terms of socialization in children, the school environment is the space par excellence (Wentzel and Looney, 2007). Therefore, in this situation, emphasis should also continue to be placed on promoting active relationships, with schools playing a primary role in the development and well-being of children.

Further, the children also report feeling happy and cheerful being at home with their family, because now they have more options to spend time and play with their parents, brothers, and sisters. This indicates the great work that families are doing to create safe and pleasant spaces, even in adverse situations like this, particularly in nurturing resilience in children exposed to epidemics (Jiao et al., 2020). Resilience is an attribute that helps children to manage everything from minor disappointments to major life traumas (Goldstein and Brooks, 2005). Amid the current COVID-19 crisis, research from China suggests that resilience should be nurtured by public health programs implemented by healthcare professionals, schools, and families in order to help children to overcome conditions of distress, and prospectively provide them with emotional and psychological support (Pettoello-Mantovani et al., 2019; Dalton et al., 2020; Jiao et al., 2020)

Further, given the results of our lexical similarity analysis, it is worth noting that we again observed the appearance of the emotions of fear, nervousness, sadness, happiness, calmness, boredom, and anger. Some of these emotions, particularly those linked to fear, sadness, worry, or nervousness, have already been identified in other studies (Jiloha, 2020), but new emotions have also emerged here. In particular, emotions of anger and boredom need to be considered as they have been noted as risk factors for mental health during lockdown (Brooks et al., 2020) and have already appeared in previous lockdown experiences during the SARS epidemic (Cava et al., 2005). In addition, the fact that these emotions are represented in relation to schoolwork should be analyzed more carefully, since it might need to be considered whether this work is an additional source of conflict for families, as certain pedagogues point out (Tonucci, 2020).

Finally, there appears to be one particular question that repeatedly comes to the minds of the children, especially to the oldest ones, that is, when is this situation going to finish? It is clear that this question cannot be answered by anyone at this time, but this call for answers also makes it obvious that children need to be considered in communications regarding COVID-19. In fact, several academics have argued that communication about the epidemic in both family and institutional networks is essential for mitigating its effects and is also one of the best tools for fostering resilience (Dalton et al., 2020; Jiloha, 2020; Weaver and Wiener, 2020).

It is worth noting that this research also has some limits that should be mentioned. First of all, the main limitation refers to the way in which data were collected, that is through parents. Although this choice was due to lockdown circumstances, the presence of parents may have altered some responses, especially those of younger children. Secondly, the sample of this research includes a range of children of very varied ages, from 3 to 12 years. And although the results have pointed to some differences among the responses of children from different ages, their understandings for an epidemic disease and for their own cognitions and feelings probably will vary quite differently.

In short, we are experiencing an unprecedented and rapidly changing situation. Understanding the emotional patterns linked to the current pandemic from the voice of those that are most vulnerable i.e., children, and identifying how they cognitively represent and emotionally face this new situation could help to lay bare the strategies that could be developed in order to help them deal with the crisis from a psychological, emotional, and social sphere. To begin with, this research has shown that, contrary to popular belief, children are not impervious to COVID-19. They are experiencing this health crisis and its consequences first-hand, and they are feeling the considerable effects of these unprecedented circumstances at different levels – not only emotionally, but also in physical and social terms. Special attention must also be paid to the emotions of fear, worry, guilt, loneliness, boredom, and anger, with an emphasis on strengthening resilience and offering psychological support to parents and children, a point that has already been emphasized by a number of scholars during this crisis (Coyne et al., 2020). In this regard, it will be essential for governments and local authorities to develop social and inclusive policies that address the psychological, social, health, and well-being needs of children, which could help to mitigate the possible effects that they could suffer as a consequence of this crisis.

## Data Availability Statement

The data that support the findings of this study are available on request from the corresponding author. The data are not publicly available due to their containing information that could compromise the privacy of research participants.

## Ethics Statement

The studies involving human participants were reviewed and approved by Ethics Committee of the UPV/EHU was obtained [M10/2020/055]. Written informed consent to participate in this study was provided by the participants’ legal guardian/next of kin.

## Author Contributions

All authors listed have made a substantial, direct and intellectual contribution to the work, and approved it for publication.

### Conflict of Interest

The authors declare that the research was conducted in the absence of any commercial or financial relationships that could be construed as a potential conflict of interest.

## Footnotes

^1^Idoiaga, N., Berasategi, N., Eiguren, A., and Dosil, M. (2020a). Education as an intervention strategy against COVID-19: social representations and emotions. (Under review).

^2^Idoiaga, N., Berasategi, N., and Ozamiz, N. (2020b). Coping with COVID-19: social representations and emotions. (Under review).
